# Influence of Alcohol Consumption on Adherence to and Toxicity of Antiretroviral Therapy and Survival

**Published:** 2010

**Authors:** R. Scott Braithwaite, Kendall J. Bryant

**Keywords:** Alcohol consumption, human immunodeficiency virus (HIV), HIV treatment, antiretroviral therapy, medication adherence, immunosuppression

## Abstract

Antiretroviral therapy (ART) has substantially altered the fate of HIV-infected people, transforming the infection from an invariably fatal disease to a chronic condition manageable by pharmacotherapy. However, in order for ART to be effective, patients must adhere strictly to an often-demanding treatment regimen. Alcohol consumption may impact survival of HIV-infected patients through a variety of pathways. Some of these are not related to the effectiveness of ART (e.g., alcohol-induced immunosuppression that exacerbates the HIV-related immunosuppression, increased hepatotoxicity, and increased mortality from non–HIV-related causes). However, some pathways mediating alcohol’s negative effect on survival are related to ART effectiveness. In particular, alcohol consumption may reduce adherence to ART, leading to decreased ART effectiveness and, ultimately, increased HIV-related mortality. Both clinical data and computer simulations have yielded information about the impact of alcohol consumption on medication adherence in both HIV-infected and noninfected patients. The findings suggest that alcohol-related nonadherence may account for a substantial amount of preventable mortality among HIV-infected patients. These findings may have clinical implications with respect to optimal treatment for HIV-infected patients who also consume alcohol.

Combination antiretroviral therapy (ART)—highly potent therapy “cocktails” of three or more antiretroviral drugs (ARVs)—has transformed HIV care. Prior to the advent of ART in the late 1990s, infection with the human immune deficiency virus (HIV) was viewed as a “death sentence.” Thus, in 1995, the standardized annual mortality rate was approximately 500 per 1,000 person-years (Hooshyar et al. 2007), and acquired immune deficiency syndrome (AIDS) was the most prevalent cause of death among individuals aged 25–44 in the United States (Centers for Disease Control and Prevention 1994). Following the introduction and dissemination of combination ART, deaths from HIV infection plummeted to one-fifth of their prior level (i.e., 100 per 1,000 person-years in 2002) (Hooshyar et al. 2007). This sharp reduction in HIV mortality suggests that approximately 80 percent of deaths in HIV-infected individuals are being prevented or postponed through the availability of ART. As a result, HIV infection has metamorphosed into a chronic disease manageable with pharmacotherapy.

Although HIV-related deaths have declined dramatically from their peak in the pre-ART era, preventable HIV-related deaths still are common among the many patients who do not adhere strictly to their ARV medications. ART typically involves a complex regimen of several pills that must be taken at different times during the day, and to ensure its long-term effectiveness, at least 95 percent of drug doses need to be taken exactly as directed (Paterson et al. 2000). However, studies found that only 39 percent to 91 percent of ART doses are taken as directed (Braithwaite et al. 2007; Chesney et al. 2000), resulting in reduced treatment effectiveness. Alcohol consumption is a strong and consistent risk factor for poor ART adherence across a wide spectrum of patient cohorts and care settings (Cook et al. 2001; Lucas et al. 2002; Samet et al. 2004; Tucker et al. 2003). This article explores the pathways through which alcohol consumption may impact survival among HIV-infected people, particularly those related to reduced ART adherence.

## Impact of Alcohol Consumption on HIV Mortality

Alcohol consumption may increase mortality among HIV-infected patients through many pathways. Some of these are mediated by alcohol-induced changes in ART effectiveness, whereas others are independent of ART effectiveness. This article emphasizes pathways mediated through changes in ART adherence, because the high proportion of HIV deaths preventable by ART suggests that ART adherence has a substantial effect on preventable mortality and morbidity. However, the following sections first will take a look at non–ART-related pathways of alcohol’s effects.

### Pathways Independent of ART Effectiveness

Alcohol consumption may influence survival of HIV-infected patients without altering the effectiveness of individual ARVs or the entire ART regimen. These effects may be mediated by three general mechanisms:

- Increasing HIV-related mortality directly (e.g., by exacerbating immunosuppression);
- Enhancing the toxicity of ART (e.g., by exacerbating the toxic effects of ARVs on the liver [i.e., hepatotoxicity]); and
- Increasing mortality unrelated to HIV or ART (e.g., by accelerating liver damage from concurrent infection with the hepatitis C virus [HCV] or increasing the likelihood of traumatic injury).

#### Increased Immunosuppression

Data from laboratory studies (i.e., in vitro studies), animal models, and human studies suggest that alcohol consumption may increase HIV-related mortality directly by inducing immunosuppressive effects that are additive to or synergistic with those of HIV. For example, in in vitro studies, alcohol exposure inhibited the normal immune responses of lymphocytes from AIDS patients (Nair et al. 1994). In other studies, alcohol consumption by HIV-infected patients increased the multiplication (i.e., replication) of the virus in another type of white blood cells called mononuclear cells (Bagasra et al. 1993, 1996; Balla et al. 1994). Finally, alcohol treatment of isolated lymphocytes and other blood cell lines enhanced HIV infection of these cells (Wang et al. 2006). Several animal models also have suggested that alcohol can exacerbate HIV-related immunosuppression. In a rhesus macaque model of HIV, alcohol consumption at binge-drinking quantities increased viral replication (Poonia et al. 2006*a*) and altered the distribution and cycling of different lymphocyte subsets in a manner that resulted in an impaired cellular immune response (Poonia et al. 2006*b*). Moreover, in a mouse model, high levels of alcohol ingestion caused increased inflammation of brain tissue by increasing the concentration of HIV-infected immune cells in the brain (Potula et al. 2006). Finally, in another rhesus macaque model, chronic binge alcohol consumption increased the likelihood that an opportunistic infection would occur (which corresponds to clinical AIDS in humans), even if the alcohol-exposed and control animals exhibited the same level of immunosuppression (Bagby et al. 2006). In humans, alcohol consumption has been found to reduce the production of immune molecules (i.e., antibodies) in response to vaccination and has been associated with a greater decline in the number of those lymphocytes that constitutes the primary targets of HIV infection (i.e., CD4 cells) prior to ART initiation (Samet et al. 2007).

#### Amplified ART Toxicity

Many ARVs have substantial side effects, including hepatoxicity. Although few studies have directly evaluated the association between alcohol consumption and ARV toxicity, there is plausible basis for concern. Through its metabolic pathways, alcohol may act synergistically with other hepatotoxic processes to increase the magnitude of hepatic inflammation and steatosis and thus also may magnify the side effects of ARVs (Lieber 2003, 2004; Montaner et al. 2003; Ogedegbe and Sulkowski 2003; Rockstroh and Spengler 2004). One of the main metabolic pathways for alcohol in the liver is the microsomal ethanol-oxidizing system (MEOS), which primarily is mediated through a family of molecules called cytochrome P450 (Lieber 2003, 2004). Heavy alcohol consumption may lead to excessive activation of the MEOS, which in turn may contribute to the generation of reactive oxygen species and oxidative stress. These processes play a role in the development of steatosis and hepatitis. This pathway of MEOS activation and increased oxidative stress increasingly is implicated as a cause of liver failure, even when factors other than alcohol consumption are responsible for its initiation (i.e., in cases of nonalcoholic steatohepatitis [NASH]). The hepatotoxicity of ARVs (e.g., zidovudine, didanosine, stavudine, and nevirapine) also is mediated through this pathway and contributes to the syndrome of mitochondrial injury that is a well-known potential consequence of certain ART regimens and which is characterized by nausea, jaundice, lactic acidosis, and lipoatrophy.

Because alcohol consumption and ARVs both impact the MEOS pathway, alcohol consumption has the potential to greatly exacerbate the side effects from ART. Furthermore, ART-related dysfunction of the MEOS may decrease the effectiveness with which alcohol is metabolized and consequently may increase the magnitude and duration of intoxication after alcohol consumption.

#### Pathways Unrelated to HIV Infection

Because ART has lowered HIV-related mortality, a greater proportion of deaths among HIV-infected patients now can be attributeed to non–HIV-related causes. In particular, liver failure has become an increasingly important cause of death in this population, accounting for 13 percent of all deaths among HIV-infected people and 24 percent of non–AIDS-related deaths (Rosenthal et al. 2007). Many HIV-infected people in the United States and Europe are coinfected with HCV, and alcohol consumption amplifies the risk that this infection will cause cirrhosis. Accordingly, alcohol consumption likely causes many deaths from liver failure among people who are coinfected with HIV and HCV. Indeed, in a large French cohort, nearly all (93 percent) deaths from liver failure in HIV patients were sequelae of HCV infection, and in the vast majority (70 percent) alcohol consumption was a contributing factor (Rosenthal et al. 2007).

It is important to note that often overlap and synergy exist between ART-dependent and ART-independent pathways for hepatic toxicity. Thus, activation of the MEOS pathway from ART toxicity most likely occurs among those HIV-infected people who already have MEOS damage at baseline for other reasons (e.g., HCV infection) or who exhibit biochemical evidence (i.e., serological markers) suggestive of MEOS damage (i.e., altered levels of certain liver enzymes called transaminases).

### Pathways Dependent on ART Effectiveness

The impact of alcohol consumption on survival of HIV-infected patients likely also is mediated by pathways affecting patient adherence to the ART regimens and, ultimately, ART effectiveness. In fact, these pathways likely play a particularly crucial role because patients must take at least 95 percent of their ART medication doses correctly to ensure enduring ART effectiveness (Paterson et al. 2000). Alcohol consumption is a strong and consistent risk factor for poor ART adherence, and some of this impact may be mediated by the alcohol-related amplification of ART toxicity—that is, patients may choose to skip ART doses after consuming alcohol because they are afraid of toxic symptoms or of combined hepatotoxic effects. In the short-term, nonadherence is the primary cause of ART failure as has been shown in numerous patient groups and disparate health care settings (Haubrich et al. 1999; Le et al. 2001; Lucas et al. 1999; Nieuwkerk et al. 2001; Phillips et al. 2001). For example, in a two-site U.S. cohort, increases in the number of virus particles in the blood (i.e., virological failure) occurred only in 22 percent of patients with adherence rates of 95 percent or more but in 80 percent of those with adherence rates of less than 80 percent (Lepri et al. 2000). Similarly, a study of a large Swiss cohort found that in 56 percent of patients who exhibited increases in virus particle numbers after an initial reduction (i.e., who exhibited viral load rebound), this rebound was preceded by nonadherence (Paris et al. 1999). In the longer term, nonadherence not only leads to virological failure but also increases the likelihood that the virus becomes resistant to different ARVs, thereby lowering the effectiveness of ART (Bangsberg et al. 2000; Tam et al. 2008). Reduced effectiveness, in turn, amplifies the likelihood that the patients’ infection will progress to AIDS. Thus, Lima and colleagues (2008) estimated that among HIV-positive people starting ART, those with moderate adherence (i.e., 40 percent to 80 percent of doses taken as directed) were 3.6 times more likely to develop AIDS compared with people with at least 95 percent adherence and those with poor ART adherence (i.e., less than 40 percent of doses taken as directed) were 5.9 times more likely to develop AIDS.

### Estimating the Effect of Alcohol Consumption on ART Nonadherence

As mentioned previously, alcohol consumption has been associated with nonadherence in a wide variety of settings and patient groups, with estimates of effect ranging from odds ratios^1^ of 1.7 to 4.7(Cook et al. 2001; Lucas et al. 2002; Samet et al. 2004; Tucker et al. 2003). In other words, alcohol-consuming patients were 1.7 to 4.7 times more likely to exhibit nonadherence than were abstainers. However, interpreting these findings has been complicated by concerns that other factors may confound the results because people who consume alcohol likely differ from abstainers in ways that are difficult to control for, even in multivariate analyses. In an attempt to reduce residual confounding, Braithwaite and colleagues (2005*a*) analyzed whether alcohol consumption on a particular day was associated with nonadherence on that same day. To this end, the investigators used a procedure called timeline followback, which involves participants keeping a retrospective daily diary of alcohol consumption (Sobell and Sobell 1992). The investigators sought to identify same-day associations with alcohol consumption, which are more suggestive of a causal association, and disaggregate them from more temporally distant associations, which are less suggestive of a causal association. To reduce the likelihood that any reporting bias exaggerated the relationship between alcohol consumption and medication adherence, separate timeline followback procedures were performed for alcohol consumption and for medication adherence. The study participants were part of the Veterans Aging Cohort Study (VACS), an eight-site observational study that was designed to isolate the effects of HIV by enrolling both HIV patients receiving care and HIV-negative control subjects who also received medical care and who were matched by age, race, and site. Because the VACS mostly enrolled patients with chronic diseases, nearly all HIV-negative control patients also were taking one or more medications. Therefore, the investigators could compare associations between alcohol consumption and medication adherence in HIV-positive and HIV-negative groups.

#### Temporal Analyses of Alcohol Consumption and Nonadherence

**Figure 1:**
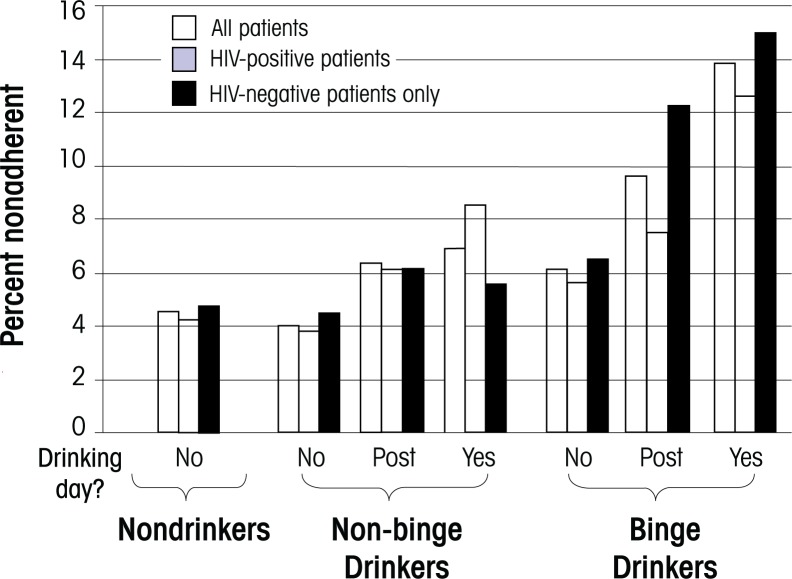
Percentage of medication nonadherence on a given calendar day among HIV-positive and HIV-negative patients with different levels of alcohol consumption. The effects of alcohol consumption on the same or a proximal day on medication adherence are shown. “No” denotes a day with no active or recent alcohol consumption, “post” denotes a day with no active alcohol consumption but with alcohol consumption on the previous day, and “yes” denotes a day of active alcohol consumption. HIV-positive patients have a lower threshold at which alcohol impacts nonadherence. SOURCE: Adapted from Braithwaite et al. 2005*a*.

Braithwaite and colleagues (2005*a*) determined that regardless of HIV status, consumption of alcohol on a particular day was associated with decreased adherence to medications on that day and on the following day (i.e., the postdrinking day), and heavier drinking was associated with an increased likelihood of nonadherence (see figure 1). Thus, the percentage of abstainers who missed medication doses on a given day was 4 percent. Among nonbinge drinkers (i.e., drinkers who consumed less than five standard drinks per day), 7 percent missed medication doses on drinking days, 6 percent on postdrinking days, and 4 percent on nondrinking days (*P* < 0.001 for trend). Among binge drinkers (i.e., drinkers who consumed five or more drinks per day), 14 percent missed doses on drinking days, 10 percent on postdrinking days, and 6 percent on nondrinking days (*P* < 0.001 for linear trend). Relationships of similar magnitude were seen when HIV-positive and HIV-negative participants were analyzed separately, except that clinically significant (i.e., at least 5 percent) increases in nonadherence were seen in the HIV-positive, but not in HIV-negative, nonbinge drinkers.

#### Threshold Effects of Alcohol Consumption on Nonadherence

**Figure 2:**
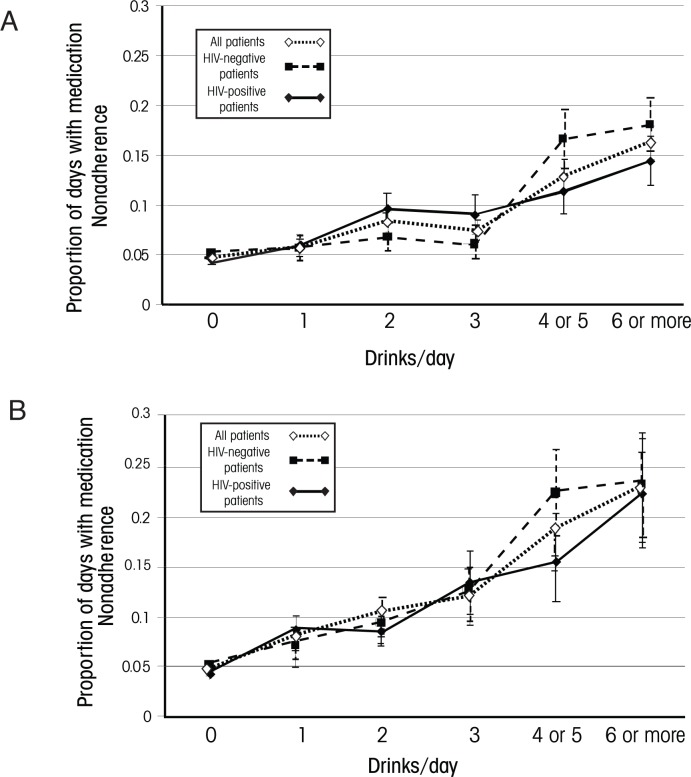
Percentage of days with medication nonadherence among people who reported consuming alcohol on the same calendar day, stratified by alcohol consumption levels. Panel A shows the data without any adjustment, whereas panel B shows the data with adjustment for each participant’s mean alcohol consumption.* Both panels provide the data for all study participants as well as for HIV-positive and HIV-negative participants separately. Adjusting results for mean daily consumption improves identification of the consumption threshold (approximately two drinks) at which clinically significant nonadherence occurs and reduces apparent differences between HIV-positive and HIV-negative participants. Thus, whereas in unadjusted analyses the threshold at which alcohol impacts adherence appears to be lower for HIV-positive patients than for HIV-negative patients, the curves for the two groups are more similar once consumption has been adjusted for mean daily alcohol consumption. SOURCE: Adapted from Braithwaite et al. 2008. *For the adjustment, the person’s mean daily quantity of alcohol consumed was subtracted from the quantity of alcohol he reportedly consumed on a particular day.

**Figure 3:**
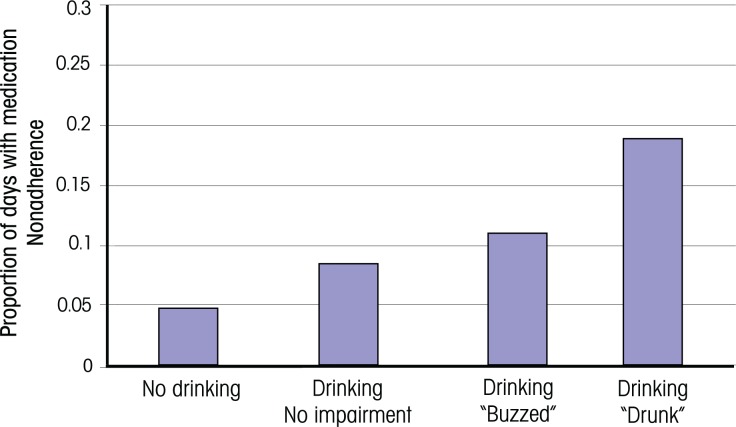
Percentage of days with medication nonadherence among study participants who had consumed alcohol on the same calendar day, with alcohol consumption classified in terms of the participants’ self-rated threshold for cognitive impairment (“none” versus “buzzed” versus “drunk”). Clinically significant nonadherence occurred at drinking levels that resulted in participants reporting feeling “buzzed.” SOURCE: Braithwaite et al. 2008.

Because nonadherence effects in HIV-infected people were substantial even among nonbinge drinkers, a follow-up study sought to determine whether moderate quantities of daily alcohol consumption were associated with nonadherence in this population and whether a particular threshold was identifiable for this effect (Braithwaite et al. 2008). In addition, the investigators assessed whether these results were impacted if daily alcohol consumption was adjusted for individual-specific factors, such as a person’s usual alcohol consumption quantity and threshold for cognitive impairment (e.g., feeling “buzzed” or “drunk”). The study found that in HIV-positive patients, adherence showed a clinically significant decline (i.e., 5 percent or more) at a threshold of two standard drinks. This threshold was substantially lower than that of HIV-negative participants (i.e., four standard drinks) (figure 2). When the data were adjusted for each person’s mean daily alcohol consumption,^2^ however, the differences between HIV-positive and HIV-negative participants were reduced, and in both groups clinically significant nonadherence was observed at thresholds of two or more standard drinks. Similarly, when each participant’s daily alcohol consumption was compared with his self-reported threshold for impairment, results also were comparable for HIV-positive and HIV-negative participants (figure 3). Thus, in both groups clinically significant nonadherence occurred at drinking levels that resulted in the participants feeling “buzzed” or “drunk.” Considered together, these results raise the possibility that any impact of HIV and/or ART on the threshold at which alcohol consumption induces nonadherence may be mediated through more irregular alcohol consumption patterns and/or a lower threshold for intoxication.

#### Estimating the Impact of Alcohol Consumption–Induced ART Nonadherence on Survival

In another study, Braithwaite and colleagues (2007*b*) used a validated computer simulation of HIV survival (Braithwaite et al. 2005*b*, 2006, 2007*a*) to estimate how ART nonadherence associated with alcohol consumption would be expected to impact survival. The investigators used the simulation to “sum” this effect over time and to weigh it against competing risks of death that are unrelated to HIV. Separate analyses were conducted for different daily consumption levels (i.e., nonbinge and binge drinking) and for different frequencies of consumption (i.e., once weekly, weekend pattern [two or three times per week on consecutive days], and daily drinking).

Based on the previous analysis of VACS data (Braithwaite et al. 2005*a*), the relative risk of nonadherence associated with alcohol consumption was 1.5 on nonbinge drinking days and 2.7 on binge drinking days. When these relative risks were applied to the lowest baseline nonadherence rate that was consistent with VACS data (i.e., a rate of 5 percent, which assumed that self-report did not lead to any systematic underreporting of nonadherence), daily nonbinge alcohol consumption decreased survival from 29.2 years to 27.0 years, which corresponds to an 8 percent decrease. Moreover, under these assumptions, daily binge alcohol consumption decreased survival from 28.1 years to 21.4 years, a 24 percent decrease. Finally, binge alcohol consumption decreased survival by at least 1 year at all frequencies of once weekly or greater.

The investigators also applied the same relative risks to the highest baseline nonadherence rate that was consistent with VACS data (i.e., a rate of 25 percent), which assumes that self-report led to systematic underreporting of nonadherence and uses an alternative baseline nonadherence estimate based on pharmacy refill data. Using these assumptions, drinking in nonbinge quantities at any frequency of once per week or greater diminished median survival by more than 1 year. At a frequency of twice per week, nonbinge drinking reduced survival by 2.1 years (from 21.7 years to 19.6 years), and with daily non-binge drinking, survival was reduced by 3.3 years (from 21.7 years to 18.4 years), a 15 percent decrease. With binge drinking, the effects were even more pronounced. Thus, binge drinking at any frequency of once per week or greater diminished median survival by more than 2 years, and at a frequency of twice per week, it reduced survival by 4.0 years (from 16.1 years to 12.1 years). Finally, with daily binge drinking, survival was reduced by 6.4 years (from 16.1 years to 9.7 years), a 40 percent decrease. (Note that binge drinkers started off with a lower life expectancy than nonbinge drinkers in these analyses [16.1 years rather than 21.7 years] because they were less adherent with ART overall, even on days when they were not drinking.)

It is important to keep in mind that these analyses still may have underestimated the impact of alcohol consumption on survival because they only consider one particular pathway through which alcohol consumption may lower survival (i.e., enhanced nonadherence), although this mechanism likely is particularly important. In addition, the analyses factored in at least one pathway through which alcohol consumption may decrease mortality (i.e., the J-curve association, in which moderate alcohol consumption may lower death from cardiovascular causes). Furthermore, it should be noted that any inference regarding the impact of alcohol consumption on survival involves assumptions about causality that cannot be established by observational analysis, regardless of methodological sophistication.

Several other related issues also still need to be addressed. For example, the design of the studies did not allow the investigators to identify particular psychosocial mediators that may underlie increased nonadherence with alcohol consumption past a certain threshold. For example, patients may forget to take medications after drinking, may deliberately skip medications after being told not to take them with alcohol, or may fear that taking their medications will reveal their HIV status in social settings. Moreover, the investigators did not examine whether the threshold is different for medication regimens with reduced pill burdens (e.g., one pill a day).

### Summary of the Effect of Alcohol Consumption on ART Nonadherence

Epidemiological and computer simulation results suggest that alcohol consumption has a consistent and substantial association with levels of ART nonadherence, resulting in premature morbidity and mortality in HIV patients. The threshold at which alcohol consumption impacts medication adherence may be lower in HIV-infected people than in noninfected people, especially for those patients who have fluctuating levels of consumption or who are at increased risk for hepatoxicity or cognitive impairment.

## Implications for Clinical Care

**Figure 4:**
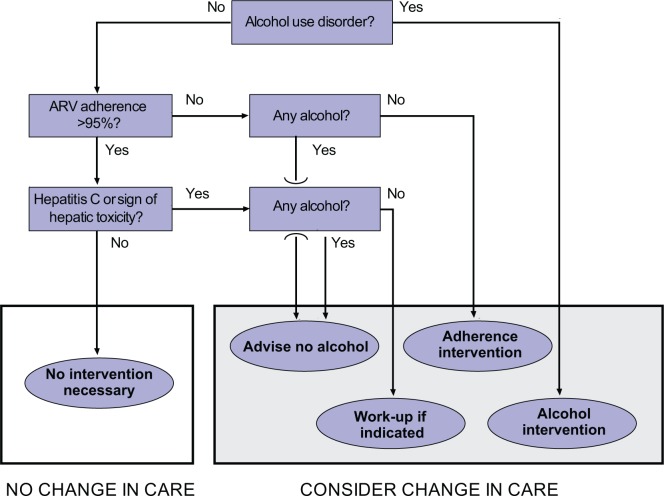
Example of an algorithm for integrating alcohol screening and treatment into clinical care of HIV patients. Several possible algorithms are supported by current data. As health informatics and clinical decision supports become more commonplace in clinical care, algorithms such as this one have increasing potential to influence how care is delivered.

Although it may be premature to propose changes in clinical care algorithms based on these limited data, it is instructive to consider how such results may lead to improvements in care if confirmed by more definitive follow-up studies. In such a scenario, each HIV-infected patient could be screened on a regular basis for hazardous alcohol consumption and other alcohol use disorders (see figure 4). If a patient screens positive, then he or she could receive an intervention directed at reducing unhealthy alcohol consumption.

If a patient screens negative for alcohol use problems, the next step would be to ascertain medication adherence levels to determine whether adherence is sufficiently low (e.g., less than 95 percent) to lead to short-term or long-term decrement in ARV effectiveness. If the setting of care includes an electronic medical record system with information on pharmacy refills, adherence can be approximated using one of several existing validated algorithms (Steiner et al. 1988). If such a system is not available, adherence levels can be approximated via validated self-report questions, although in this case underreporting is an important consideration. If ART adherence is determined to be less than 95 percent, the patient could be asked whether he consumes any alcohol, because even subhazardous levels of alcohol consumption are associated with poorer adherence. Alcohol-consuming patients then could be advised to refrain from any alcohol consumption.

Alcohol-consuming patients with ART adherence of 95 percent or higher could be assessed for HCV infection and/or signs of hepatotoxicity. This is most commonly done by reviewing the most recent set of laboratory data that are routinely obtained for people on ART and which include liver function tests. If these tests indicate presence of HCV or signs of liver toxicity, the patient could be advised to cease all alcohol consumption. If there is no sign of HCV or liver toxicity, then no change in care would be necessary.

## Future Work

Although possible directions for future work are extraordinarily diverse (Bryant 2006), three areas are particularly important. First, it is crucial to generate more experimental evidence that can inform the care of HIV-infected patients with alcohol use disorders. This should include randomized controlled trials in HIV-infected people of alcohol interventions that have been successful in other populations (e.g., pharmacotherapy such as topirimate or naltrexone, brief motivational interventions) (Johnson et al. 2007). Such trials will be essential in order to assess the impact of such interventions on alcohol consumption, ART adherence, ART effectiveness, and progression to liver failure.

Second, future work should explore the impact of alcohol consumption on HIV transmission. This article only has described the relationship between alcohol consumption and mortality among people already infected with HIV; however, alcohol consumption likely also plays an important role in facilitating transmission of the virus to as yet uninfected people. Third, as electronic medical records and decision supports become more commonplace, it will be important to anticipate how research insights may be translated into clinical care improvements. In particular, clinical decision supports can integrate algorithms such as the one presented in figure 4, helping clinicians to decide when an alcohol intervention is appropriate, to choose the most appropriate intervention, and to prioritize alcohol interventions within the context of other important clinical issues based on rank order of expected benefit or other objective criteria.

## Conclusion

Alcohol consumption even at non-binge levels (e.g., two or more standard drinks per day) may impact survival in HIV-infected people through numerous pathways. One of the most important pathways likely is mediated through effects on ART adherence. Additional research is needed to better characterize the strength and causality of these relationships and to use them to inform clinical care.

## Footnotes

1 An odds ratio is a measure of effect size. It is defined as the ratio of the odds of a particular outcome (e.g., medication non-adherence) in patients exposed to a certain factor (e.g., alcohol consumption) and patients who were not exposed to that factor.

2 Adjustment was achieved by subtracting each person’s mean daily quantity of alcohol consumed from the quantity of alcohol he consumed on a particular day.
